# Inhibition of Monoacylglycerol Lipase Suppresses Lipopolysaccharide-Enhanced Melanoma Proliferation and Migration

**DOI:** 10.3390/cancers18203255

**Published:** 2026-10-09

**Authors:** Faris Almutairi, Yazeed Alneef, Hussam Albassam, Saad Alobid, Ahlam M. Alhusaini, Dalal Alkhelb, Abdullah S. Alhamed, Mohammed Alqinyah, Ahmed Altuwaijri, Sabry M. Attia

**Affiliations:** Department of Pharmacology and Toxicology, College of Pharmacy, King Saud University, Riyadh 11451, Saudi Arabia; 447100447@student.ksu.edu.sa (Y.A.); halbassam@ksu.edu.sa (H.A.); saaalobaid@ksu.edu.sa (S.A.); aelhusaini@ksu.edu.sa (A.M.A.); dalkhelb@ksu.edu.sa (D.A.); asalhamed@ksu.edu.sa (A.S.A.); malqinyah@ksu.edu.sa (M.A.); aaltuwaijri@ksu.edu.sa (A.A.); attiasm@ksu.edu.sa (S.M.A.)

**Keywords:** melanoma, monoacylglycerol lipase, 2-arachidonoylglycerol, lipopolysaccharide, toll-like receptor 4, cyclooxygenase-2, migration, proliferation

## Abstract

Background/Objectives: Lipid metabolism dysregulation contributes to cancer progression. Monoacylglycerol lipase (MAGL) breaks down 2-arachidonoylglycerol (2-AG) into glycerol and arachidonic acid, a substrate for cyclooxygenase-2 (COX-2) to produce pro-inflammatory mediators such as prostaglandin E2 (PGE2). Lipopolysaccharide (LPS) activation of Toll-like receptor 4 (TLR4) triggers inflammatory pathways, including COX-2/PGE2 signaling, implicated in diseases such as melanoma. MAGL inhibition reduces prostaglandins and other inflammatory mediators, but its role in inflammation-driven melanoma progression remains unexplored. This study investigated how JZL184, a selective MAGL inhibitor, and 2-AG affect LPS-induced inflammation, proliferation, and migration in B16 melanoma cells. Methods: Cells received LPS alone or with JZL184 or 2-AG. We measured inflammatory transcripts by qRT-PCR, COX-2 protein by Western blot, PGE2 release by ELISA, proliferation by CCK-8, and migration by scratch assay. Results: LPS increased COX-2, PGE2, *VEGF*, *N-cadherin*, and inflammatory cytokine expression. JZL184 suppressed these LPS-induced changes and reduced cell proliferation and migration. In contrast, 2-AG did not alter LPS-induced proliferation or inflammatory signals. JZL184’s effects on inflammatory gene expression and proliferation were independent of CB1 receptor signaling, suggesting its anti-melanoma activity may result from reduced inflammatory mediators rather than enhanced endocannabinoid signaling. Conclusions: These findings provide initial evidence that MAGL may regulate inflammation-triggered melanoma progression and could be a plausible therapeutic target.

## Simple Summary

Dysregulated lipid metabolism may drive melanoma progression by worsening inflammation. This study tested whether MAGL inhibition with JZL184 or treatment with the endocannabinoid 2-arachidonoylglycerol (2-AG) could reduce lipopolysaccharide (LPS)-induced inflammation and tumor growth. B16 melanoma cells received LPS alone or with 2-AG or JZL184, with or without AM 251. We measured inflammatory markers, including COX-2 and PGE2, along with cell proliferation and migration. LPS increased COX-2, PGE2, *VEGF*, *N-cadherin*, and inflammatory cytokines, while promoting cell proliferation and migration. JZL184 reduced these inflammatory responses and inhibited growth and migration. In contrast, 2-AG did not counter LPS effects. JZL184’s inhibition of LPS-induced inflammatory gene expression and cell proliferation was independent of CB1 signaling. These findings suggest that JZL184’s anti-melanoma effects in B16 cells may result from reduced inflammatory mediators rather than enhanced endocannabinoid signaling by 2-AG.

## 1. Introduction

Melanoma remains the leading cause of skin cancer-related deaths and continues to significantly affect patients, despite advances in diagnosis and treatment [1,2,3]. To improve patient prognosis, innovative therapies are needed to target the signaling pathways involved in melanoma growth, migration, invasion, and chemoresistance.

Lipid metabolism dysregulation is a feature of cancer progression, affecting tumorigenic properties through altered lipid synthesis or degradation [4,5]. Lipid hydrolysis is regulated by various enzymes, with monoacylglycerol lipase (MAGL) serving as the rate-limiting enzyme in monoacylglycerol (MAG) catabolism, releasing glycerol and free fatty acids (FFAs) [6]. These FFAs are precursors to pro-tumorigenic signaling lipids, including phosphatidic acid (PA), lysophosphatidic acid (LPA), and prostaglandin E2 (PGE2), which promote cell proliferation, growth factor production, neovascularization, migration, metastasis initiation, and chemotherapy resistance [7,8]. MAGL mainly hydrolyzes 2-arachidonoylglycerol (2-AG). This monoacylglycerol activates cannabinoid receptors 1 and 2, producing glycerol and arachidonic acid, which serves as a precursor for various inflammatory mediators, including PGE2 [9].

The MAGL activity was significantly increased across several models of human aggressive cancer cells [10,11]. In correlation with the significant increase in MAGL activity, higher levels of FFAs were found in a variety of aggressive cancer cells compared to non-aggressive cancer cells. More importantly, inhibition of the enzymatic activity of MAGL with JZL184 (a selective inhibitor of MAGL) or using short hairpin RNAs (shRNAs) against MAGL upregulated several MAGs, including 2-AG, and downregulated various corresponding FFAs in these aggressive cancer cells [10,11]. The significant reduction in FFAs leads to dramatic decreases in oncogenic signaling lipids, including LPA and PGE2, which could mediate the tumorigenic features of MAGL by promoting proliferation, migration, and invasion in these aggressive cell lines and xenograft tumor growth in murine models [10,11,12,13,14,15,16].

In addition to its role in cancer, MAGL is highly expressed in the brain and immune cells, where its inhibition provides important anti-inflammatory and protective effects in multiple disease models with underlying chronic inflammation [8]. MAGL regulates arachidonic acid synthesis, an essential substrate for cyclooxygenase-2 (COX-2) to produce pro-inflammatory mediators, including PGE2. Both COX-2 and PGE2 are key contributors to inflammation in various diseases, including melanoma, making therapeutics targeting this pathway effective [17]. Therefore, targeting MAGL signaling is a functional strategy to abolish arachidonic acid/COX-2/PGE2 signaling, which is implicated in cancer progression and other pathologies.

Inhibition of MAGL decreases microglial activation, the release of cytokines, and the neurotoxic effects of lipopolysaccharide (LPS), suggesting that it could serve as a promising drug target for a range of neuroinflammatory and neurodegenerative disorders [18,19,20,21,22,23,24]. Interestingly, treatment of hippocampal neurons with exogenous 2-AG or elevation of endogenous 2-AG by MAGL inactivation inhibited NF-κB activity, cytokines, and COX-2 expression [25]. These studies indicate that MAGL acts as a key regulator of inflammatory signaling either by enhancing the anti-inflammatory effects of 2-AG-dependent activation of cannabinoid receptors or by diminishing levels of free fatty acids, including arachidonic acid, and of pro-inflammatory arachidonic acid-derived prostaglandins and other mediators.

Numerous studies have supported the link between inflammation and cancer progression [17,26]. Inflammation is a crucial contributor to melanoma progression, and inflammatory mediators, including cytokines and oncogenic lipids, have also been implicated in progression [27,28]. Toll-like receptor 4 (TLR4) is expressed in various cancer cells, including melanoma cells, and is linked to multiple signaling pathways involved in cell proliferation and survival [27]. Stimulation of TLR4 by LPS, the classical activator of TLR4, in a subset of melanoma cells induces expression of numerous pro-inflammatory genes, including COX-2 [29]. Abundant evidence suggests that TLR4 activation following LPS treatment contributes to melanoma progression [30,31,32,33,34].

Given the critical biological role of MAGL as an inflammatory regulator, along with its elevated expression levels in aggressive melanoma cells, the main goal of this study is to elucidate the role of the MAGL pathway in regulating LPS-induced inflammatory signals in melanoma cells and to assess its impact on inflammation-driven proliferation and migration.

## 2. Materials and Methods

### 2.1. Cells

The melanoma cell lines CHL-1 (human) and B16 (mouse), and the mouse macrophage cell line RAW 264.7 were obtained from the American Type Culture Collection. CHL-1, B16, and RAW 264.7 cells were cultured in Dulbecco’s modified Eagle’s medium (Thermo Fisher Scientific, Waltham, MA, USA, cat. no.11965092) supplemented with 10% fetal bovine serum (Thermo Fisher Scientific, Waltham, MA, USA, cat. no. 10270-106) and antibiotics (1% penicillin/streptomycin) and incubated at 37 °C in a humidified atmosphere of 5% CO_2_.

### 2.2. Reagents

Lipopolysaccharide (LPS) (cat. no. HY-D1056), monoacylglycerol lipase inhibitor JZL184 (cat. no. HY-15249), 2-Arachidonoylglycerol (cat. no. HY-W011051), and AM 251 (cat. no. HY-15443) were purchased from MedChemExpress (Monmouth Junction, NJ, USA).

### 2.3. Quantitative Real-Time Polymerase Chain Reaction

Cell RNA extraction was performed using TRIzol reagent (Invitrogen, Carlsbad, CA, USA, cat. no. 15596018), and the isolated RNA was treated with DNase to remove residual genomic DNA contamination prior to reverse transcription. Subsequently, 2 μg of total RNA was used for cDNA synthesis using the High-Capacity Reverse Transcriptase cDNA kit (Applied Biosystems, Foster City, CA, USA, cat. no. 4368814) per the standard protocol. Following cDNA synthesis, each sample was diluted 10-fold, and 5 µL of the diluted cDNA was used in a 20 µL PCR reaction containing SYBR Green qPCR Master Mix (Low ROX; MedChemExpress, Monmouth Junction, NJ, USA, cat. no. HY-K0522) and primers at a concentration of 10 µM each. The mRNA expression levels were normalized to the housekeeping GAPDH gene, and the fold change was calculated using the 2^−ΔΔCT^method. Mouse and Human primers for gene amplification were obtained from Integrated DNA Technologies. The primer sequences are provided in Table 1.

### 2.4. Western Blotting

Treated cells were rinsed twice with ice-cold phosphate-buffered saline (PBS) and lysed in RIPA buffer supplemented with a protease/phosphatase inhibitor cocktail (Cell Signaling Technology, Danvers, MA, USA, cat. no. 5872S). Protein concentration was determined using the Pierce™ BCA Protein Assay Kit (Thermo Fisher Scientific, Waltham, MA, USA, cat. no. 23225). The cell lysates were mixed with 2X SDS-PAGE sample buffer and boiled for 10 min at 95 °C, and 20 µL of the protein sample was loaded onto 12% SDS-PAGE gels for separation. Subsequently, the gels were transferred to nitrocellulose membranes (Bio-Rad, Hercules, CA, USA, cat. no. 1620115) using a standard protocol. The Blotted membranes were blocked with 5% non-fat dry milk and incubated overnight with the primary antibodies: rabbit anti-COX-2 (1:1000, ABclonal Biotechnology, Woburn, MA, USA, cat. no. A3560) and rabbit anti-GAPDH (1:3000, ABclonal Biotechnology, Woburn, MA, USA, cat. no. AC001). Following primary antibody incubation, the blotted membranes were washed three times with 1X TBS-T buffer and then incubated at room temperature for one hour with the appropriate secondary HRP-conjugated antibody, goat anti-rabbit IgG-HRP (1:5000, ABclonal Biotechnology, Woburn, MA, USA, cat. no. AS014). Following three washes with 1X TBS-T buffer, Super Signal™ West Pico PLUS Chemiluminescent Substrate (Thermo Fisher Scientific, Waltham, MA, USA, cat. no. 34580) was used to detect HRP immunoreactivity. ImageJ Software (version 1.54g) was used to quantify Western blot bands, which were then normalized to the endogenous control, GAPDH, and expressed as a fold change relative to the control.

### 2.5. PGE2/Prostaglandin E2 Enzyme-Linked Immunosorbent Assay (ELISA)

Culture supernatants that were collected from cells following LPS treatment with or without the JZL184 inhibitor were centrifuged at 250 g for 10 min at 4 °C to remove cellular debris and then assayed for PGE2 levels using PGE2 ELISA (ABclonal Biotechnology, Woburn, MA, USA, cat. no. RK09052) according to the manufacturer’s instructions. Briefly, PGE2 standards or samples were applied to the plate for two hours, then incubated with the detection antibody for two hours, followed by streptavidin-HRP for 20 min at room temperature. The plate was washed with 0.05% Tween^®^ 20 in PBS between steps. The plate was visualized with TMB substrate, incubated with stop solution, and then analyzed on a microplate photometer at 450 nm (EPOCH2, BioTek Instruments, Inc., Winooski, VT, USA).

### 2.6. General 2-Arachidonoylglycerol ELISA Kit

Culture supernatants collected after vehicle or JZL184 treatment were centrifuged at 250 g for 10 min at 4 °C to remove debris, then assayed for 2-AG using 2-AG ELISA (ABclonal Biotechnology, Woburn, MA, USA, cat. no. RK00604) according to the manufacturer’s instructions.

### 2.7. Wound Healing Assay

Cell monolayers were grown in 6-well plates until they reached confluence. Using a sterile 200 µL pipette tip, a single straight-line scratch was made in each well, and floating cells and cell debris were washed away with PBS. The cells were then treated with the designated agents for 24 h. At zero time post-scratch and after 24 h of treatment, images of the scratched area in the cells were captured using a 4X objective on an EVOS XL Core microscope. The rate of migrated cells in the scratched area was calculated using ImageJ software (version 1.54g).

### 2.8. Cell Proliferation Assay

Approximately 10 × 10^3^ cells in 100 µL of cell culture medium were seeded in a 96-well plate. The next day, the cells were treated with the designated agents for 48 h. 10 µL of the cell proliferation agent CCK-8 solution containing WST-8 [2-(2-methoxy-4-nitrophenyl)-3-(4-nitrophenyl)-5-(2,4-disulfophenyl)-2H-tetrazolium, monosodium salt] (MedChemExpress, Monmouth Junction, NJ, USA, cat. no. HY-K0301) was added to each well, and the cells were then incubated in a CO_2_ incubator for 3 h in the dark. The absorbance was measured at 450 nm using a microplate reader (BioTek, Winooski, VT, USA, Elx-800).

### 2.9. Statistical Analysis

All quantified data were compiled from three independent experiments with technical replicates performed where applicable, and analyzed for statistical differences between groups using Student’s *t*-test or one-way ANOVA followed by Tukey post hoc analysis. Prism software was used to carry out statistical analyses. Data are presented as mean ± SEM. A *p*-value less than 0.05 indicates statistical significance, marked by * (compared to control cells) or # (compared to LPS-treated cells).

## 3. Results

### 3.1. The Effect of LPS Treatment on COX-2 and TNF-α Expression in B16 and CHL-1 Cells

**Figure 1:**
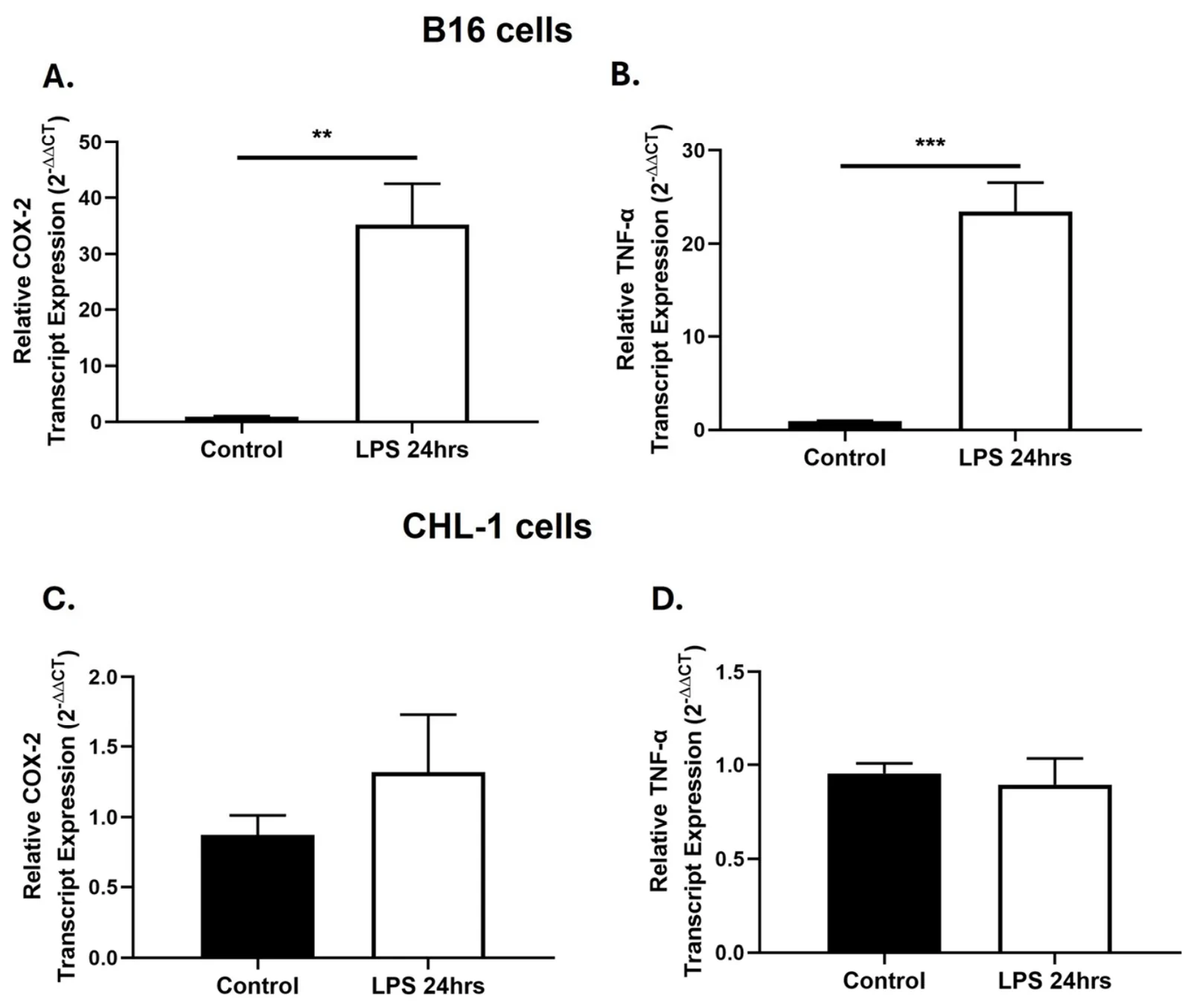
The effect of LPS treatment on *COX-2* and *TNF-α* expression in B16 and CHL-1 cells. B16 cells or CHL-1 cells were plated in a 6-well plate, adhered overnight, and were either untreated (control) or treated with LPS (10 μg/mL) for 24 h. The levels of *COX-2* and *TNF-α* in B16 cells (**A**,**B**) and CHL-1 cells (**C**,**D**) were measured by quantitative RT-PCR and normalized to the endogenous housekeeping gene *GAPDH*. Data were analyzed for statistical differences using a Student’s two-tailed unpaired *t*-test between groups. Data are presented as mean ± SEM, where **: *p* < 0.01 and ***: *p* < 0.001 indicate significance levels relative to control.

To assess the extent of the response to LPS in two distinct melanoma cell line models, we treated the cells with LPS (10 µg/mL) for 24 h. Following this treatment, we measured mRNA levels of COX-2 and tumor necrosis factor-alpha (TNF-α). Our results showed significantly heightened *COX-2* and *TNF-α* gene expression levels in B16 cells in response to LPS stimulation (Figure 1A,B). In contrast, we did not observe a similar trend for *COX-2* and *TNF-α* transcripts in CHL-1 cells following LPS treatment (Figure 1C,D). Thus, B16 cells were selected for the subsequent experiments because of a strong inflammatory response to LPS.

### 3.2. The Impact of MAGL Inhibition by JZL184 on 2-AG Level in B16 Cells

**Figure 2:**
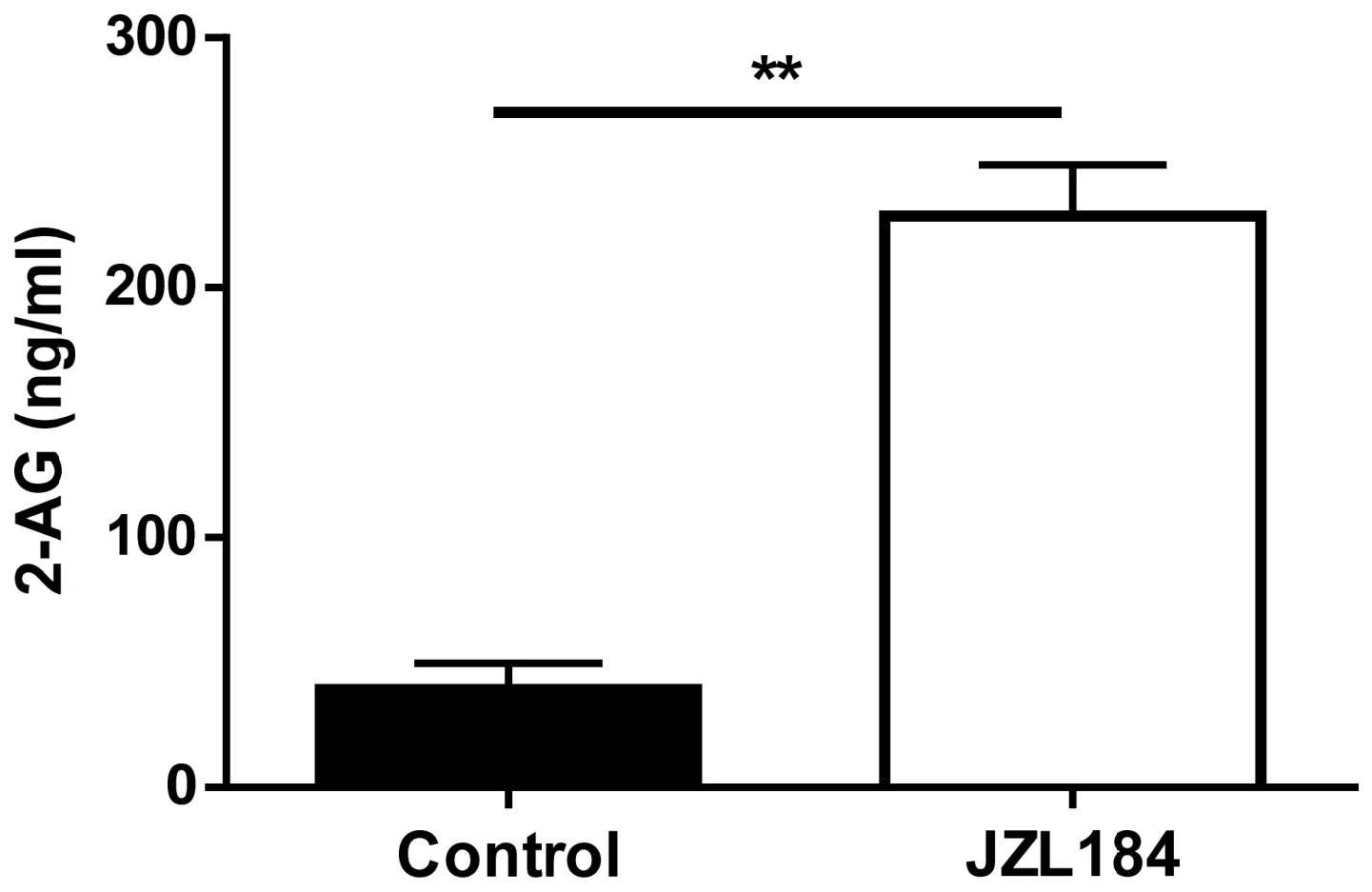
The impact of MAGL Inhibition by JZL184 on 2-AG level in B16 cells: Cells were untreated (control) or incubated with JZL184 (5 μM) for 24 h. Culture medium was collected to measure 2-AG levels using ELISA. Data were analyzed for statistical differences using a Student’s two-tailed unpaired *t*-test between groups. Data are presented as mean ± SEM, where **: *p* < 0.01 indicates significance levels relative to control.

MAGL is a key enzyme involved in the metabolism of 2-arachidonoylglycerol (2-AG). To confirm that pharmacological inhibition of MAGL by JZL184 modulates 2-AG levels in B16 cells, cells were treated with or without JZL184 (5 μM), and 2-AG concentrations in the culture medium were subsequently quantified by ELISA. Treatment with JZL184 significantly increased 2-AG levels compared with untreated control cells, consistent with inhibition of 2-AG degradation by MAGL (Figure 2).

### 3.3. The Impact of MAGL Inhibition on the LPS-Triggered COX-2/PGE2 Signaling Pathway

**Figure 3:**
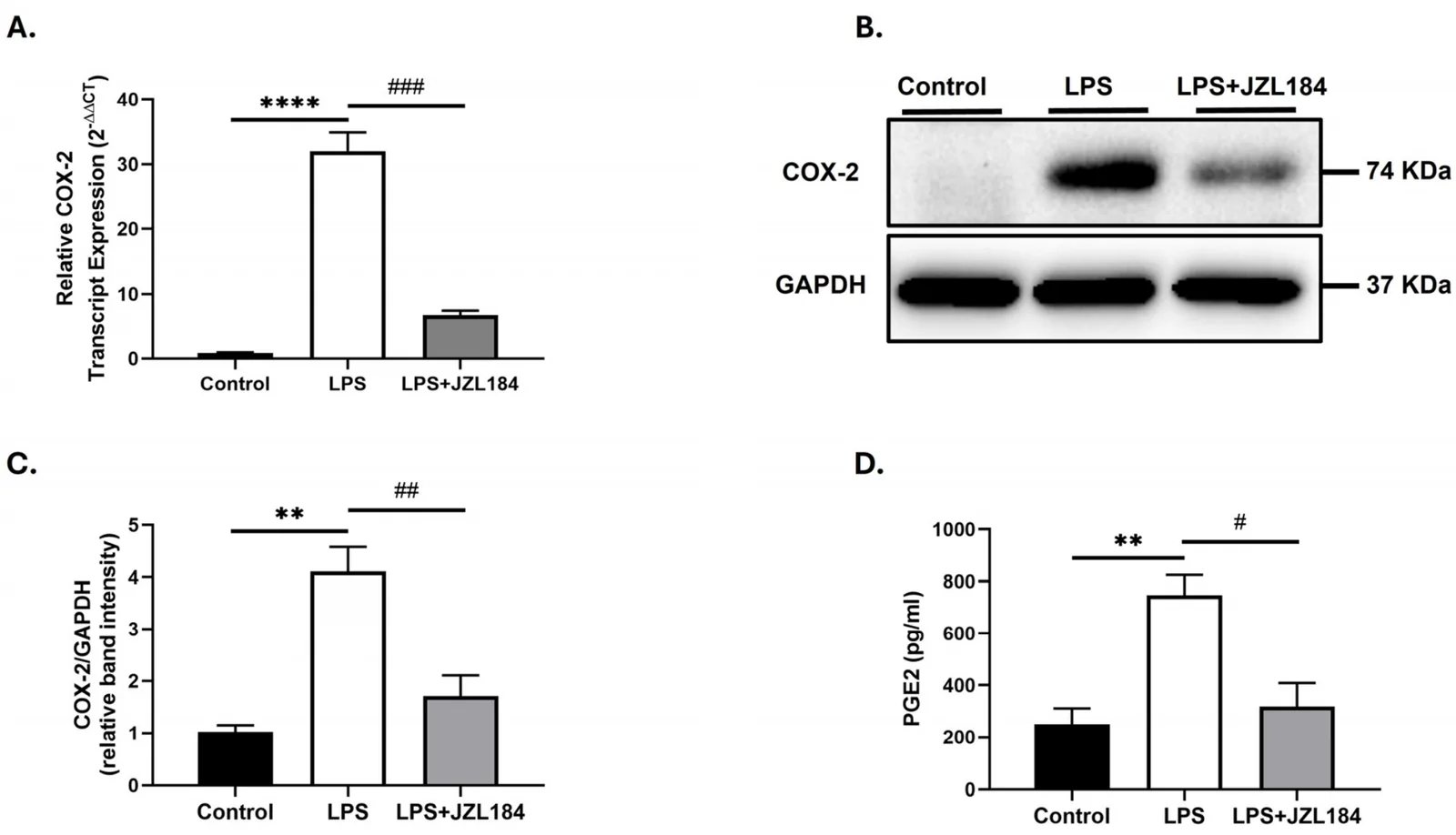
The impact of MAGL inhibition on the LPS-triggered COX-2/PGE2 signaling pathway. B16 cells were seeded in a 6-well plate and allowed to adhere overnight. Cells were untreated (control), incubated with LPS (10 μg/mL), or incubated with LPS (10 μg/mL) plus JZL184 pretreatment (5 μM) for 24 h. (**A**) *COX-2* transcript levels were measured by qRT-PCR and normalized to the endogenous housekeeping gene *GAPDH*. (**B**) The cells were lysed and subjected to SDS-PAGE and immunoblotting using specific antibodies against COX-2 and GAPDH. (**C**) Densitometry of the COX-2 band was normalized to GAPDH. (**D**) A culture medium was collected to measure PGE2 levels using ELISA. Fold differences in qRT-PCR, immunoblotting densitometry, and ELISA data were calculated following normalization to the control condition and were subjected to statistical analysis using one-way ANOVA followed by the Tukey post hoc test for group comparisons. All representative data are pooled from three independent experiments and are presented as mean ± SEM, where #: *p* < 0.05; ** or ##: *p* < 0.01; ###: *p* < 0.001 and ****: *p* < 0.0001.

To assess the impact of inhibiting MAGL on the activation of the COX-2/PGE2 pathway induced by LPS, we pretreated B16 cells with the MAGL inhibitor JZL184 (5 μM) for one hour, followed by LPS stimulation (10 µg/mL) for 24 h, and measured *COX-2* transcript and protein levels. Our findings demonstrated that stimulation of B16 cells with LPS significantly increased the mRNA and protein levels of COX-2 (Figure 3A–C). However, MAGL inhibition by JZL184 downregulated the induction of COX-2 in response to LPS at both the mRNA (Figure 3A) and protein levels (Figure 3B,C) (Figure S1). COX-2 plays a crucial role in synthesizing prostaglandins, particularly PGE2, which serves as a critical mediator for the progression of multiple cancer cells [35]. Therefore, we next used ELISA to quantify PGE2 secretion in the B16 culture medium in the presence of LPS with or without the JZL184 inhibitor. Co-treatment of B16 cells with LPS and the JZL184 inhibitor produced significantly lower PGE2 levels than those treated with LPS alone (Figure 3D). Altogether, these data indicate that MAGL activity is required for LPS-dependent COX2/PGE2 signaling pathway activation.

### 3.4. Evaluating the Effect of JZL184 Treatment on LPS-Induced Elevation of Inflammatory Genes

**Figure 4:**
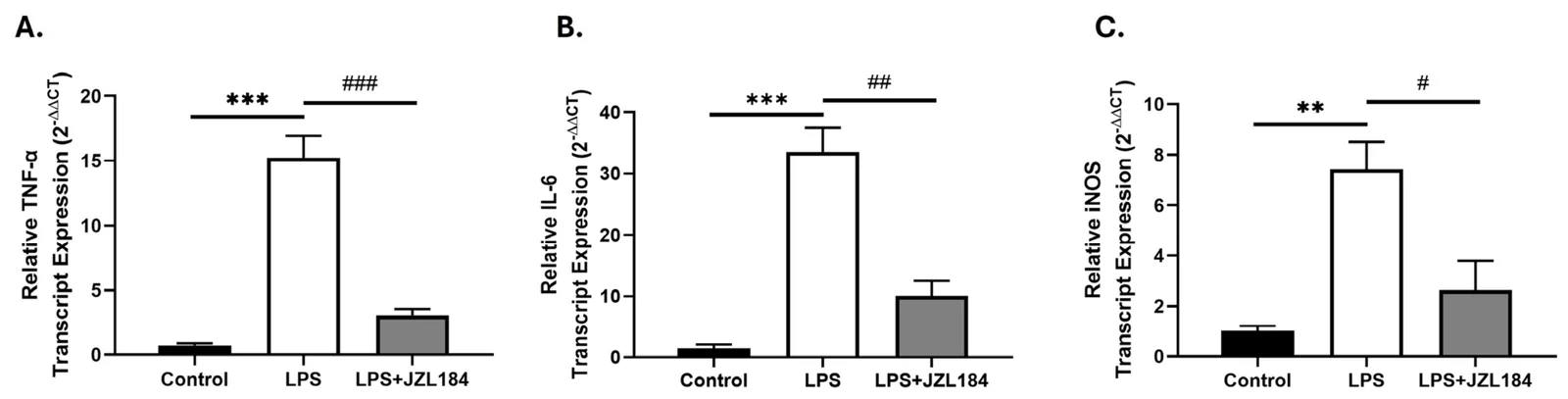
Evaluating the effect of JZL184 treatment on LPS-induced elevation of inflammatory genes. B16 cells were cultured in a 6-well plate and were then left untreated (control) or exposed to either LPS (10 μg/mL) alone or to both LPS (10 μg/mL) and JZL184 pretreatment (5 μM) for 24 h. (**A**) *TNF-α*, (**B**) *IL-6*, and (**C**) *iNOS* mRNA levels were quantified by qRT-PCR and normalized to the endogenous housekeeping gene *GAPDH*. Fold differences between groups were calculated after normalization to the control and analyzed using one-way ANOVA followed by the Tukey post hoc test. Data are presented as mean ± SEM, where #: *p* < 0.05; ** or ##: *p* < 0.01 and *** or ###: *p* < 0.001.

To further examine the effect of MAGL inhibition on additional inflammatory genes, we analyzed the effect of the JZL184 inhibitor on *TNF-α*, interleukin-6 (IL-6), and inducible nitric oxide synthase (iNOS) expression in B16 cells following LPS exposure. Similarly to the observed effect with *COX-2*, LPS induced the expression of *TNF-α*, *IL-6*, and *iNOS* transcripts, and this effect was significantly suppressed in the presence of JZL184 (Figure 4A–C). Thus, our results suggest the involvement of MAGL in regulating other LPS-induced inflammatory genes.

### 3.5. The Effect of MAGL Inhibition on LPS-Induced Proliferation and Migration of B16 Cells

**Figure 5:**
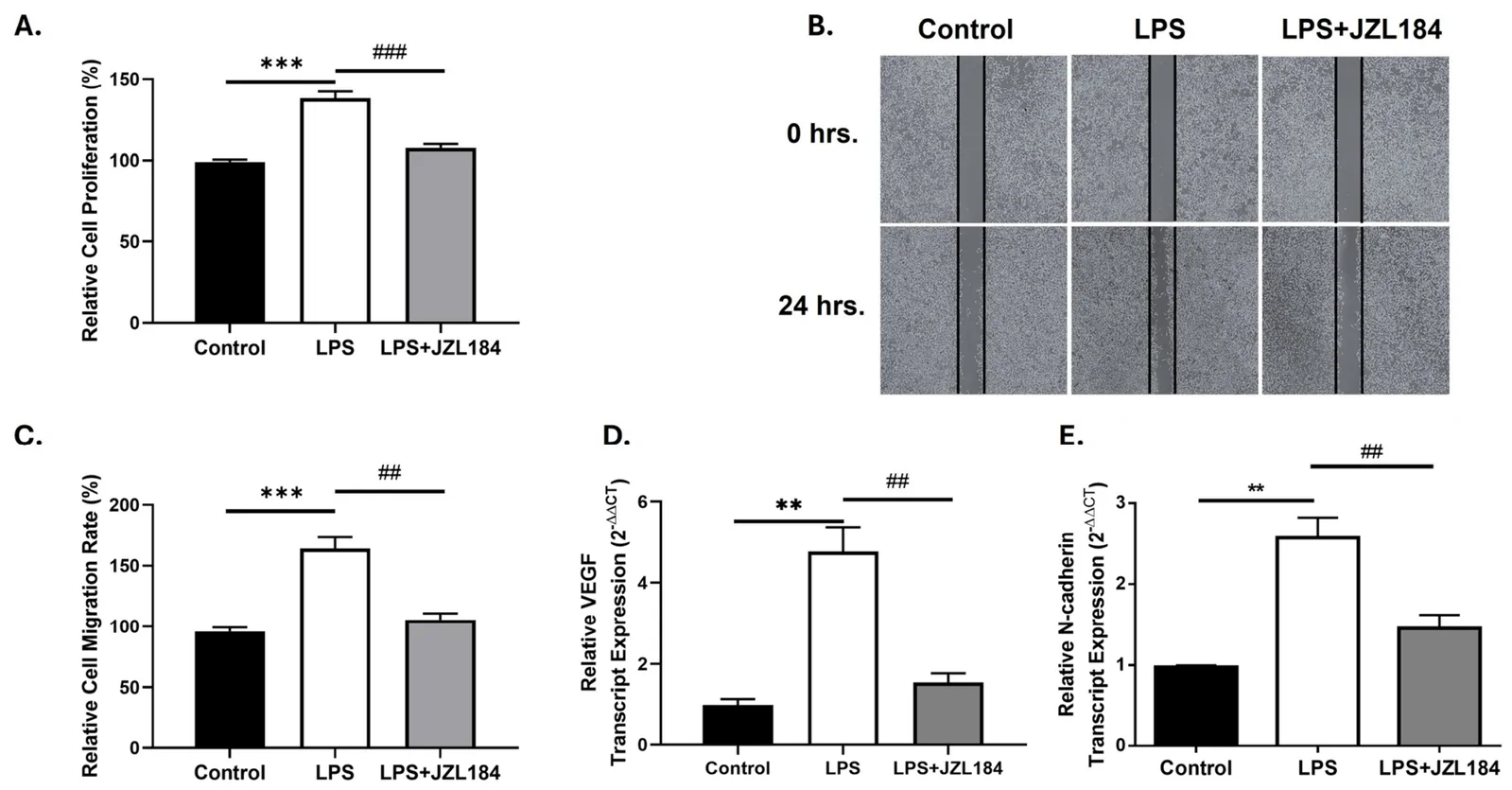
The effect of MAGL inhibition on LPS-induced proliferation and migration of B16 cells. (**A**) 10 × 10^3^ of B16 cells were untreated (control), treated with LPS (10 μg/mL), or pretreated with JZL184 (5 μM) before LPS exposure for 48 h. Cell proliferation was assessed using a CCK-8 assay. B16 cells underwent a scratch assay after 24 h of the same treatments. (**B**) Scratch images were captured at 0 and 24 h. (**C**) The bar graph shows migration rates. (**D**,**E**) B16 cells were cultured overnight in 6-well plates and then untreated (control), treated with LPS (10 μg/mL), or pretreated with JZL184 (5 μM) before LPS exposure for 24 h. *VEGF* (**D**) and *N-cadherin* (**E**) transcript levels were measured by qRT-PCR and normalized to the endogenous housekeeping gene *GAPDH*. Fold differences between groups were calculated after normalization to the control condition and analyzed using one-way ANOVA followed by Tukey’s post hoc test. The data are presented as mean ± SEM, where ** or ##: *p* < 0.01 and *** or ###: *p* < 0.001.

LPS treatment has been linked to a significant increase in melanoma cell proliferation and migration [32,36]. We next sought to determine whether JZL184 could hinder B16 cell proliferation and migration induction following LPS treatment. The CCK-8 assay was used to evaluate B16 cell proliferation after 48 h of LPS stimulation, with or without a one-hour pretreatment with JZL184. The results showed that LPS increased the proliferation of B16 cells; however, treatment with JZL184 reduced this effect (Figure 5A). To examine the effect of MAGL inhibition on cell migration, we performed a scratch assay to observe B16 cell migration following JZL184 administration and subsequent 24 h LPS incubation. The data indicated that the LPS treatment enhanced the migration of B16 cells, which was effectively inhibited by the JZL184 inhibitor (Figure 5B,C). We also quantified the transcripts of vascular endothelial growth factor (*VEGF*) and *N-cadherin* after JZL184 administration, followed by 24 h of LPS treatment. The LPS treatment significantly amplified the expression of *VEGF* and *N-cadherin* mRNA levels in the B16 cells, and the JZL184 treatment reversed the upregulation of *VEGF* and *N-cadherin* caused by LPS (Figure 5D,E). Overall, our data demonstrate that MAGL contributes to LPS-enhanced proliferation and migration of B16 cells.

### 3.6. The Effect of 2-AG Treatment on LPS-Induced Inflammatory Genes and Cell Proliferation

**Figure 6:**
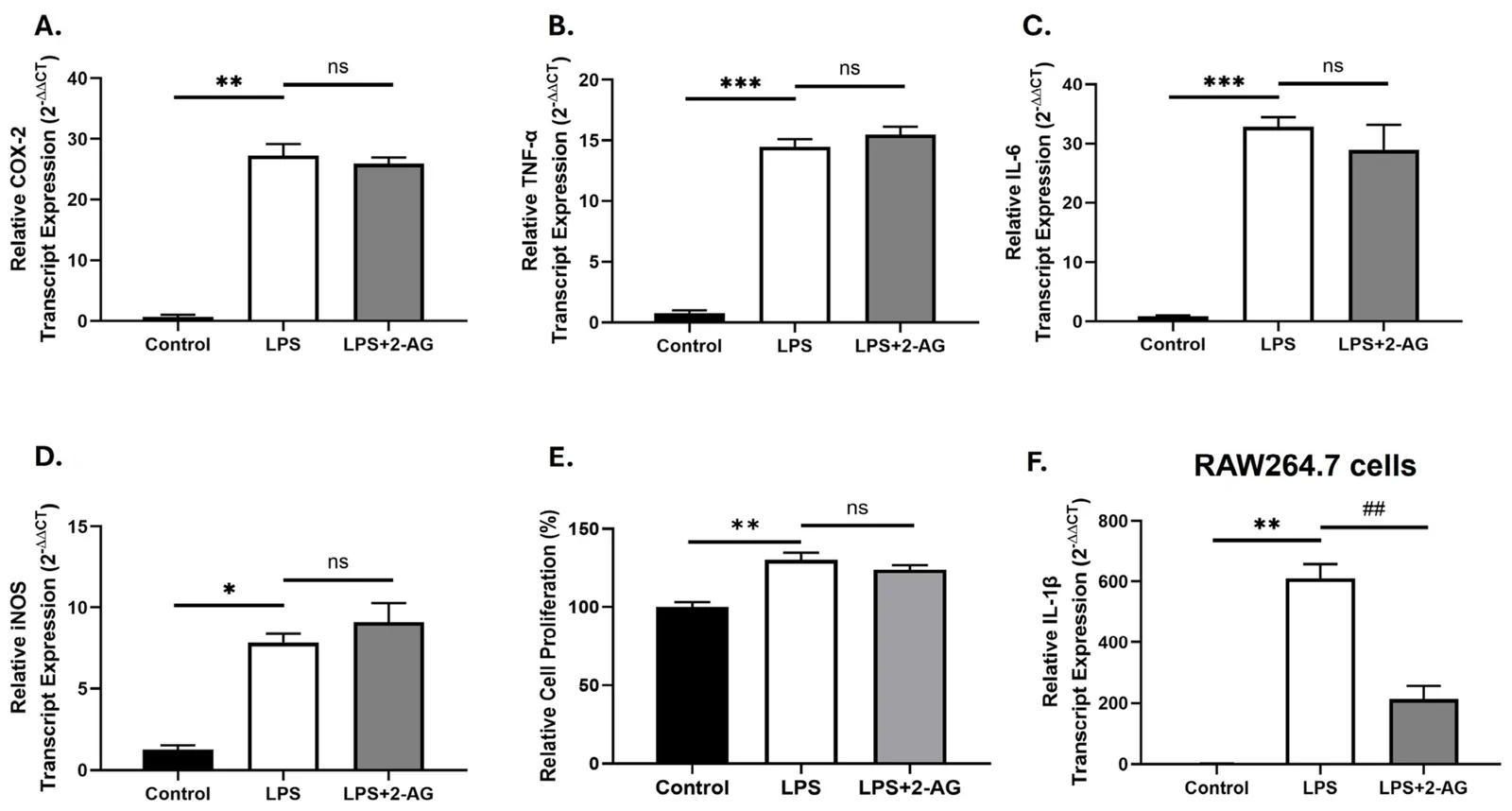
The effect of 2-AG Treatment on LPS-induced inflammatory genes and cell proliferation: B16 cells were cultured overnight in 6-well plates and left untreated (control) or treated with LPS (10 μg/mL), alone or with 2-AG (10 μM), for 24 h. Transcript levels of (**A**) *COX-2*, (**B**) *TNF-α*, (**C**) *IL-6*, and (**D**) *iNOS* were measured by qRT-PCR and normalized to *GAPDH*. (**E**) For the proliferation assay, 10 × 10^3^ of B16 cells were seeded in 96-well plates and treated with LPS (10 μg/mL), alone or with 2-AG pretreatment (10 μM), for 48 h. Proliferation was then assessed using a CCK-8 assay. (**F**) RAW264.7 cells were plated in a 6-well plate and left untreated (control) or exposed to LPS (100 ng/mL) alone, or both LPS (100 ng/mL) and 2-AG pretreatment (10 μM) for 6 h. The *IL-1β* mRNA level was measured by qRT-PCR and normalized to the endogenous housekeeping gene *GAPDH*. Fold differences between groups were calculated after normalization to the control and analyzed using one-way ANOVA followed by Tukey’s post hoc test. Data are presented as mean ± SEM, *: *p* < 0.05; ** or ##: *p* < 0.01, ***: *p* < 0.001, and ns: non-significant.

2-AG is hydrolyzed by the enzyme MAGL, contributing to the initiation of inflammatory responses [8,37]. In B16 cells, the inhibition of MAGL by JZL184 has been shown to elevate levels of 2-AG [38]. It has been found that upregulation of 2-AG, either through inhibiting the MAGL-hydrolyzing enzyme or administering exogenous 2-AG, can inhibit inflammatory responses in neurons and immune cells [25,39,40]. To determine whether treating B16 cells with exogenous 2-AG mimics the suppressive effects of MAGL inhibition by JZL184 on LPS-induced inflammatory mediators, we treated the cells with LPS (10 µg/mL) either alone or with pretreatment with 2-AG (10 μM). Then, we measured the level of inflammatory gene transcripts. Our results demonstrate that the increase in mRNA levels of *COX-2*, *TNF-α*, *IL-6*, and *iNOS* following LPS stimulation was not affected by 2-AG treatment (Figure 6A–D). We further investigated the impact of 2-AG on LPS-enhanced B16 cell proliferation and found that 2-AG did not affect LPS-induced proliferation in this cell line (Figure 6E). To ensure the effectiveness of the 2-AG used in this study, we preincubated the RAW264.7 macrophage cell line with 2-AG, followed by 100 ng/mL of LPS priming. As previously reported [38], we confirmed that treating RAW264.7 cells with 2-AG significantly decreased LPS-induced expression of *IL-1β* mRNA (Figure 6F). These findings collectively indicate that the observed protective effect of MAGL inhibition on B16 cells is potentially attributable to reduced production of inflammatory mediators.

### 3.7. The Effect of CB1 Receptor Antagonist on JZL184-Inhibited LPS-Induced Inflammatory Genes and Cell Proliferation

**Figure 7:**
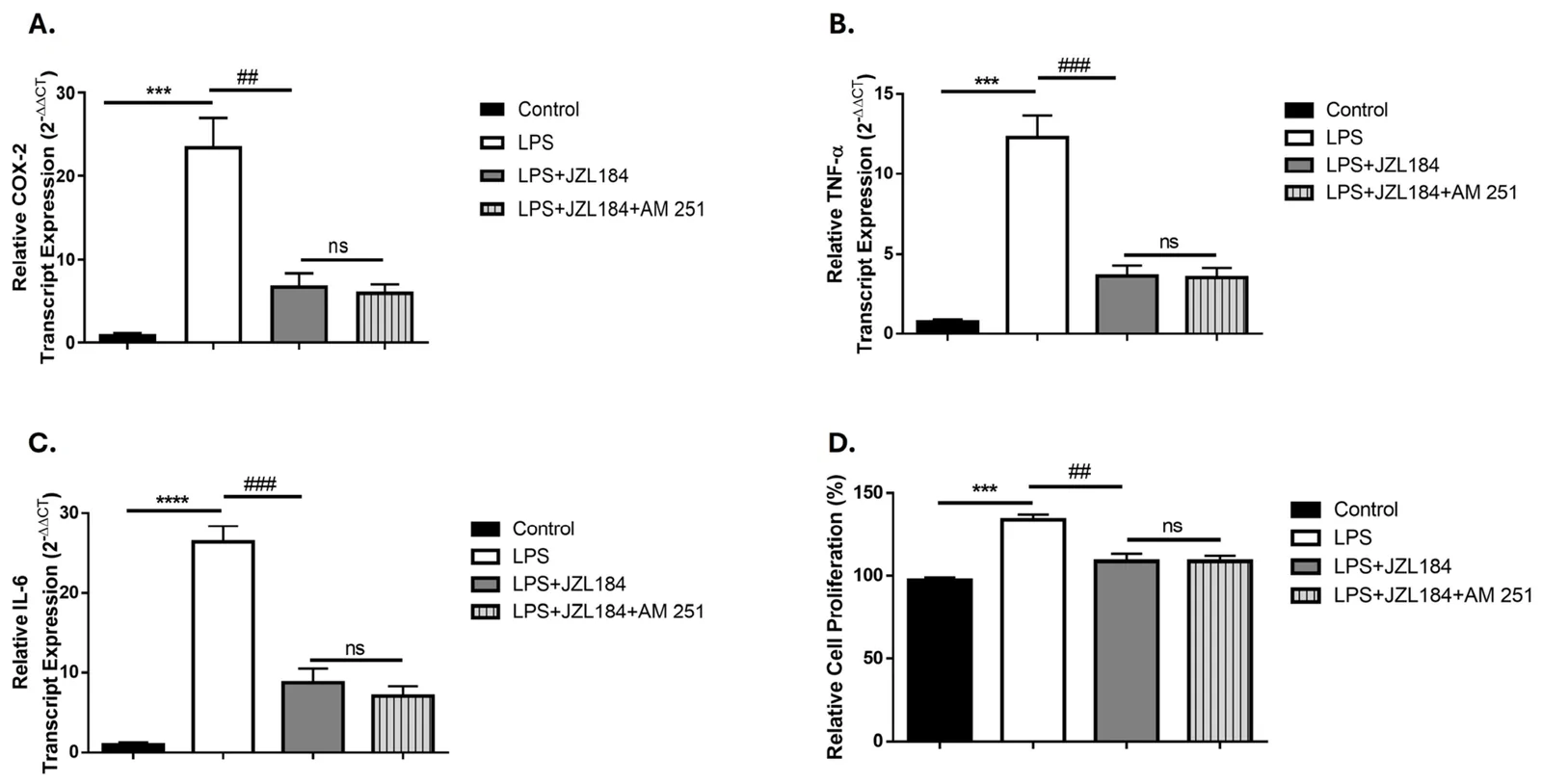
The effect of CB1 receptor antagonist on JZL184 Inhibited LPS-induced inflammatory genes and cell proliferation: B16 cells were preincubated with AM 251 (10 μM) for one hour, followed by addition of JZL184 (10 μM) and LPS (10 μg/mL) for 24 h. (**A**) *COX-2*, (**B**) *TNF-α*, and (**C**) *IL-6* mRNA levels were quantified by qRT-PCR and normalized to the endogenous housekeeping gene *GAPDH*. (**D**) 10 × 10^3^ of B16 cells were seeded in 96-well plates and treated as above for 48 h. We then assessed proliferation using a CCK-8 assay. Fold differences between groups were calculated after normalization to the control condition and analyzed using one-way ANOVA followed by Tukey’s post hoc test. The data are presented as mean ± SEM, where ##: *p* < 0.01; *** or ###: *p* < 0.001; ****: *p* < 0.0001 and ns: non-significant.

To further investigate the role of the CB1 receptor in the effects of MAGL inhibition, we assessed whether JZL184-mediated inhibition of LPS-stimulated inflammatory gene expression in B16 cells was affected by CB1 receptor blockade. We measured inflammatory gene transcripts, including *COX-2*, *TNF-α*, and *IL-6*, after JZL184 and LPS treatment, with or without pretreatment with the CB1 receptor antagonist AM 251. Interestingly, the JZL184-mediated reduction in LPS-stimulated *COX-2*, *TNF-α*, and *IL-6* mRNA levels was completely resistant to AM 251 pretreatment (Figure 7A–C). Because we previously showed that JZL184 reduced the LPS-induced increase in B16 cell proliferation, we next tested whether this effect was sensitive to AM 251 pretreatment. Consistent with inflammatory gene expression, the JZL184-mediated reduction in LPS-increased B16 cell proliferation was unaffected by AM 251 (Figure 7D). Collectively, these data suggest that JZL184-mediated MAGL inhibition reduced LPS-induced increases in inflammatory gene expression and B16 cell proliferation through a mechanism independent of CB1 receptor signaling.

## 4. Discussion

Melanoma has a significant burden for patients, even in light of substantial advancements in diagnostic and therapeutic options. Inflammation plays a pivotal role in the progression of melanoma, with various inflammatory mediators implicated in key processes such as proliferation, migration, and the development of chemoresistance [41]. Therefore, innovative therapeutic strategies that explicitly target cellular pathways to inhibit uncontrolled inflammatory signals hold great promise for effective melanoma treatment.

MAGL is a serine hydrolase, which is the key enzyme in decomposing several monoacylglycerides into glycerol and FAAs, including arachidonic acid. Arachidonic acid serves as a substrate for COX-2 to produce several pro-inflammatory mediators, including PGE2 [42]. Numerous studies have examined the role of MAGL in diseases associated with inflammation, such as Parkinson’s disease [39,43,44], multiple sclerosis [45], Alzheimer’s disease [46], rheumatoid arthritis [47,48], airway inflammation [49], and colitis [50]. Despite extensive research on MAGL in various animal and cellular models of these conditions, its biological function in inflammation-induced cancer progression, including melanoma, remains unexplored.

This study provides initial evidence suggesting that targeting MAGL may have potential for reducing inflammation and the associated proliferation and migration of melanoma cells. TLR4 belongs to the family of pattern recognition receptors that, upon activation by the bacterial epitope LPS, trigger multiple signaling pathways and subsequently release various pro-inflammatory mediators [51]. Previous studies have shown that LPS activation of TLR4 induces the production of pro-inflammatory mediators and enhances the migration and proliferation of melanoma cells, including B16 cells [30,32,36]. The elevated expression of inflammatory genes, such as IL-6, observed following LPS treatment, was consistent with previous studies reporting significantly higher expression of TLR4 in B16 cells [36,52,53]. We also assessed the effect of LPS treatment on the CHL-1 human melanoma cell line and found that it did not respond to LPS in the same manner as B16. Specifically, the production of pro-inflammatory mediators was not upregulated, suggesting differences in TLR4 expression between the two cell lines. Our findings indicate that LPS induces the production of *IL-6*, *TNF-α*, *iNOS*, *N-cadherin*, and *COX-2* transcripts in B16 cells, along with COX-2 protein and PGE2 production.

Notably, the treatment with JZL184 effectively inhibited the LPS-induced production of these pro-inflammatory mediators. These findings are consistent with previous research showing that MAGL inhibition reduces LPS-induced production of certain inflammatory mediators across various cellular and animal models [18,25,54,55]. Our findings demonstrate for the first time that inhibiting MAGL with JZL184 successfully reduced LPS-induced expression of several inflammatory genes in B16 cells.

In addition to its suppressive effects on the production of inflammatory mediators, our findings demonstrated that the MAGL inhibitor JZL184 reduced LPS-induced melanoma migration and proliferation, as well as LPS-induced *VEGF* and *N-cadherin* expression. While many studies have investigated the role of MAGL in various cancer types, such as prostate, breast, and ovarian cancer [10,11], our research specifically focused on its impact on inflammation-induced melanoma. Therefore, it will be interesting to examine the potential effects of MAGL inhibition on inflammation-driven cancer progression in other cancer types.

MAGL facilitates the hydrolysis of 2-AG, a monoacylglycerol that is an endogenous full agonist of cannabinoid receptors. Activation of these CB receptors has been widely implicated in the regulation of inflammatory and immune responses [56,57]. Additionally, 2-AG is the primary precursor for the production of proinflammatory mediators, such as prostaglandins [58]. The inhibition of MAGL activity has been demonstrated to elevate levels of 2-AG while decreasing the concentration of FFAs across various cancer cell lines, such as melanoma cells [10]. The administration of 2-AG has directly demonstrated a neuroprotective effect on hippocampal neurons against pro-inflammatory stimuli such as IL-1β, LPS, and β-amyloid, as well as excitotoxic glutamate. This protection appears to occur through the modulation of COX-2 expression and the CB1 receptor-dependent NF-kB signaling pathway [25,59,60,61,62]. 2-AG further protected neurons from LPS-induced toxicity by reducing COX-2 expression and sodium current elevation [63,64,65].

In the context of cancer, 2-AG treatment, activating the CB1 receptor, mediated the anti-invasive and antitumorigenic effect in lung cancer cells [66]. The antiproliferative effects of 2-AG have been observed in various cancer cell types [67,68,69]. However, our findings indicate that applying 2-AG did not influence the increase in inflammatory mediators and the associated proliferation following LPS treatment in B16 melanoma cells, suggesting that exogenous 2-AG application to B16 cells did not yield results comparable to those observed with JZL184. Further, we showed that JZL184-mediated MAGL inhibition reduced LPS-induced inflammatory gene expression and B16 cell proliferation through a mechanism independent of CB1 receptor signaling.

This suggests that 2-AG regulates cancer cell progression in a manner specific to the cell type. To validate the effectiveness of 2-AG, treatment of RAW264.7 cells with exogenous 2-AG for a shorter duration of 6 h resulted in a significant reduction in IL-1β levels. This discrepancy in the effect of 2-AG on inflammatory mediators in B16 cells may stem from the 24 h treatment duration, during which exogenous 2-AG may become unstable in the medium or be susceptible to hydrolysis by enzymes such as alpha/beta-hydrolase domain-containing enzyme 6 (ABHD6). Therefore, conducting additional experiments with varying treatment durations could provide valuable insights into the role of 2-AG in the context of LPS-induced inflammatory mediators and the associated proliferation in B16 cells.

Although this was not observed in lung cancer cells [66], adding free fatty acids, such as palmitic acid, has been demonstrated to reverse the anti-cancer effects of MAGL inhibition in prostate cancer cells [11]. Therefore, it would be interesting to check whether treating B16 cells with FFAs could counteract the suppressive effect of MAGL inhibitor JZL84 on LPS-induced inflammatory mediators and LPS-enhanced proliferation and migration.

In addition to MAGL, the hydrolysis of 2-AG is facilitated by ABHD6 [70,71,72]. The inhibition of ABHD6 has demonstrated protective effects against various cancer types and in cellular and murine models of diseases linked to neuroinflammation [73]. Notably, ABHD6 serves as the primary 2-AG hydrolase in macrophages, where it regulates 2-AG levels and diminishes LPS-induced prostaglandin production in vitro and in vivo [38]. Inhibition of ABHD6, using the specific inhibitor WWL70 [74], reduces acute lung inflammation by decreasing LPS-induced leucocyte recruitment. This effect is linked to lower proinflammatory cytokines and increased 2-AG levels in bronchoalveolar lavages and lung tissue [75]. Thus, future studies could explore the role of ABHD6 in melanoma progression induced by LPS, where inflammatory responses play a significant role.

This study presents valuable insights, although it is essential to acknowledge certain limitations. It was conducted using a mouse-derived cell line, specifically the B16 melanoma cell line, chosen for its high expression of MAGL and its susceptibility to LPS. To enhance the robustness of our findings, future research could benefit from examining additional types of melanoma cell lines to investigate the effects of MAGL inhibition. While the B16 cell line was the most suitable option, it would be insightful to explore human melanoma cell lines as well. Notably, CHL-1, the available human melanoma cell line, has negligible MAGL expression and is unresponsive to LPS treatment, which poses challenges for our specific objectives. Exploring cell lines with varying MAGL levels could provide a more comprehensive understanding of MAGL’s role in LPS/TLR4 activation-triggered inflammatory mediators and LPS-induced melanoma proliferation and migration. While JZL184 remains the most utilized MAGL inhibitor in research, owing to its high selectivity and irreversible action, exploring other MAGL inhibitors could be advantageous in determining whether similar findings can be achieved with alternative compounds.

## 5. Conclusions

In conclusion, the present findings suggest that MAGL contributes to LPS-induced inflammatory gene expression and the associated increases in proliferation and migration of mouse B16 melanoma cells. Pharmacological inhibition of MAGL with JZL184 attenuated these LPS-induced responses, and the effects appeared independent of CB1 receptor activation. These findings suggest that MAGL may represent a potential target for modulating inflammation-associated melanoma progression. Further studies are warranted to elucidate the underlying mechanisms and to determine the therapeutic relevance of MAGL targeting in human melanoma cells and other cancer types.

## Figures and Tables

**Table 1:** Gene primer sequences used for qRT-PCR reaction.

Gene	Forward Primer Sequence	Reverse Primer Sequence
Human primers		
*TNF-α*	GTCAACCTCCTCTCTGCCAT	CCAAAGTAGACCTGCCCAGA
*COX-2*	CCCTTGGGTGTCAAAGGTAA	GCCCTCGCTTATGATCTGTC
*GAPDH*	GCCAAGGTCATCCATGACAACT	GAGGGGCCATCCACAGTCTT
Mouse primers		
*TNF-α*	CCTGTAGCCCACGTCGTAG	GGGAGTAGACAAGGTACAACCC
*COX-2*	TGCAAGATCCACAGCCTACC	GCTCAGTTGAACGCCTTTTG
*IL-6*	AGTGGTATAGACAGGTCTGTTGG	CTGCAAGAGACTTCCATCCAG
*iNOS*	TGACGGCAAACATGACTTCAG	GCCATCGGGCATCTGGTA
*VEGF*	CATCTTCAAGCCGTCCTGTGT	CTCCAGGGCTTCATCGTTACA
*N-cadherin*	GCGCAGTCTTACCGAAGGAT	TGAATTTCACATTGAGAAGGGGC
*GAPDH*	AACTTTGGCATTGTGGAAGG	GGATGCAGGGATGATGTTCT

## Data Availability

The original contributions presented in the study are included in the article. Further inquiries can be directed to the corresponding author.

## Supplementary Materials

The following supporting information can be downloaded at https://www.mdpi.com/article/10.3390/cancers18203255/s1, Figure S1: supplemental data.

## Abbreviations

The following abbreviations are used in this manuscript:

2-AG	2-arachidonoylglycerol
ABHD6	alpha beta hydrolase domain-6
CB1R	cannabinoid receptors 1
CB2R	cannabinoid receptors 2
COX-2	cyclooxygenase-2FFAs, free fatty acids
IL-1β	interleukin-1β
IL-6	interleukin-6
iNOS	inducible nitric oxide synthase
LPA	lysophosphatidic acid
LPS	lipopolysaccharide
MAGs	monoacylglycerides
MAGL	monoacylglycerol lipase
NF-κB	nuclear factor kappa B
PA	phosphatidic acid
PGE2	prostaglandin E2
shRNA	short hairpin RNA
siRNA	small interfering RNA
TAG	triacylglycerol
TLR-4	Toll-like receptor-4
TNF-α	tumor necrosis factor-alpha
VEGF	vascular endothelial growth factor
