# Supplementary material for: Inhibition of Monoacylglycerol Lipase Suppresses Lipopolysaccharide-Enhanced Melanoma Proliferation and Migration
Source: Cancers (Basel). 2026 Oct 9;18(20):3255. doi: 10.3390/cancers18203255 (PMC13702675; doi:10.3390/cancers18203255)
Supplement: Supplementary file 1 [file cancers-18-03255-s001.zip › cancers-4477601-supplementary.pdf]

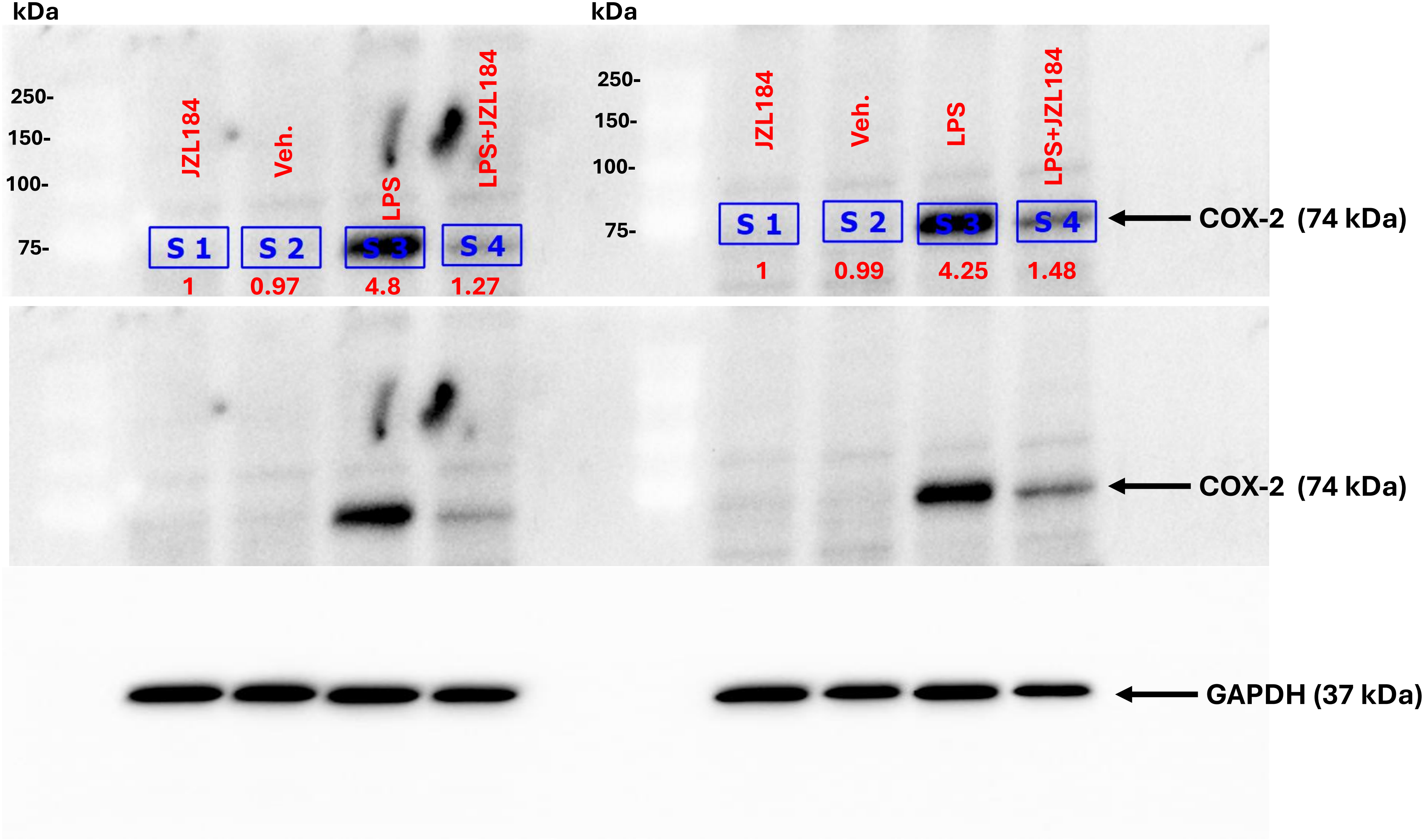

kDa
250-
150-
100-
75-
JZL184
Veh.
LPS
LPS+JZL184
S 1
S 2
S 3
S 4
1
0.97
4.8
1.27
kDa
250-
150-
100-
75-
JZL184
Veh.
LPS
LPS+JZL184
S 1
S 2
S 3
S 4
1
0.99
4.25
1.48
COX-2 (74 kDa)
COX-2 (74 kDa)
GAPDH (37 kDa)
